# Insights on the Phytochemical Profile (Cyclopeptides) and Biological Activities of *Calotropis procera* Latex Organic Fractions

**DOI:** 10.1155/2013/615454

**Published:** 2013-11-18

**Authors:** Thiago Lustosa Jucá, Márcio Viana Ramos, Frederico Bruno Mendes Batista Moreno, Mayara Patrícia Viana de Matos, José Delano Barreto Marinho-Filho, Renato Azevedo Moreira, Ana Cristina de Oliveira Monteiro-Moreira

**Affiliations:** ^1^Departamento de Bioquímica e Biologia Molecular, Universidade Federal do Ceará, Av. Humberto Monte, Campus do Pici, 60451-970 Fortaleza, CE, Brazil; ^2^Departamento de Fisiologia e Farmacologia, Faculdade de Medicina, Universidade Federal do Ceará, Avenue Coronel Nunes de Melo, 1127, Rodolfo Teófilo, 60430-270 Fortaleza, CE, Brazil; ^3^Curso de Farmácia, Universidade de Fortaleza, Avenue Washington Soares, 1321, Bloco F, Edson Queiroz, 60811-905 Fortaleza, CE, Brazil

## Abstract

*Calotropis procera* is a medicinal plant whose pharmacological properties are associated with its latex. Here, the *Calotropis procera *latex fractions were investigated in an attempt to trace its phytochemical profile and measure its anti-inflammatory and toxicity activity. The crude latex was partitioned, yielding five fractions (49.4% hexane, 5.2% dichloromethane, 2.0% ethyl acetate, 2.1% n-butanol, and 41.1% aqueous). Phytochemical screening and spectroscopy analysis revealed that dichloromethane is the most chemically diverse fraction. Triterpenes were detected in both the hexane and dichloromethane fractions, while flavonoids were detected in the dichloromethane and ethyl acetate fractions. These fractions were cytotoxic to cancer cell lines (LD_50_ 0.05 to 3.9 **μ**g/mL) and lethal to brine shrimp (LD_50_ 10.9 to 65.7 **μ**g/mL). Reduced neutrophil migration in rats was observed in carrageenan-induced peritonitis for the dichloromethane (67%), ethyl acetate (56%), and aqueous (72%) fractions. A positive reaction with tolidine and ninhydrin suggested that cyclopeptides are in the ethyl acetate fraction. It is therefore concluded that *Calotropis procera* latex dichloromethane and ethyl acetate fractions exhibit both in vitro and in vivo activities as well as anti-inflammatory properties. Cyclopeptide detection is especially interesting because previous attempts to investigate these low-molecular cyclic amino acid sequences in *C. procera* have failed.

## 1. Introduction

*Calotropis procera* is a medicinal plant and many pharmacological properties are associated with its latex, which is a rich source of biologically active compounds [1]. The efficacy of *C. procera* latex for treating inflammation-related disorders, pain, and other ailments, including neoplasia, in folk medicine has garnered scientific support [2–4]. However, the chemical composition of this latex remains under investigation. 

Latex is produced by plants in unrelated taxonomic groups, but it is most commonly found in Euphorbiaceae and Apocynaceae plants. *C. procera* is included in the later taxon. Latex is chemically diverse and the chemical and biochemical differences are considerable for different plants fluids. For instance, *Hevea brasiliensis* latex is a rich source of (poly) isoprenes and an antifungal protein (hevein) is a “fingerprint” of this latex [5, 6]. Cardenolides in the *C. procera* latex are associated with toxic effects in mammals [7]. In addition, insecticidal and antifungal proteins have been reported and their enzymatic profiles have been characterized [8, 9]. 

A series of studies support latex proteins involvement in the pharmacological properties. Although secondary metabolites have been reported in *C. procera* vegetative tissues and latex, to a lesser extent [10], limited information is available on the pharmacological properties. Fractionated latex has not been extensively investigated for phytochemical or pharmacological properties. However, the literature has demonstrated its outstanding potential for studies [1, 11]. Therefore, this study was aimed at contributing to new insights on the phytochemical profile (cyclopeptides) and biological activities of the *C. procera* latex organic fractions.

## 2. Materials and Methods

### 2.1. Reagents

Organic solvents (hexane, dichloromethane, ethyl acetate, and n-butanol) were of analytical grade. Carrageenan, Doxorubicin, glycine, potassium dichromate, and papain (E.C. 3.4.22.2) were from SIGMA Chemical Co. (São Paulo, Brazil). Tolidine and ninhydrin were from VETEC fine chemicals (Rio de Janeiro, Brazil). Trimethylsilane (TMS), deuterated chloroform, and methanol were from Tedia Brazil (Rio de Janeiro, Brazil).

### 2.2. Plant Material

The latex was collected from the aerial portions of wild plants located at Fortaleza beaches, Ceara, Brazil. A voucher specimen (n. 32663) was deposited at the Prisco Bezerra Herbarium at the local University (Universidade Federal do Ceará).

### 2.3. Extraction Method

To 200 mL of crude latex, 400 mL of distilled water was added, and the mixture was extracted successively with 600 mL of each of the following solvents: hexane, dichloromethane, ethyl acetate, and n-butanol. The extraction was repeated three times for each solvent and the resulting fractions were pooled. Each fraction was removed from the remaining mixture under reduced pressure at 40°C using an evaporator until obtaining dried fractions.

### 2.4. Qualitative Phytochemical Screening

Phytochemical tests were used to detect secondary metabolites, such as phenols, tannins, flavonoids, steroids, triterpens, saponins, and alkaloids, for each fraction in accordance with the method proposed by Matos [12]. Latex samples (25 mg/mL) were prepared in 95% ethanol and treated in an ultrasonic apparatus (30 min) prior to the measurements. The results were analyzed based on a visual observation of color modification or precipitate formation after adding specific reagents. 

Tannins and phenols were investigated by adding 60 *μ*L of 2% FeCl_3_ in ethanol to 3 mL of the latex sample. A blue-red soluble phase indicated phenols, a blue precipitated material indicated soluble tannins and a green color indicated condensed or cachectic tannins.

Three independent samples (3 mL) were prepared in tubes to test for flavonoids. Anthocyanins and antocyanidines were indicated by a red color after adding 0.1 M HCl to pH 3.0 or by a purple color by adding 0.1 M NaOH to pH 8.5 or blue-purple to pH 11.0. Flavones, flavonols, and xanthones were indicated by a yellow color at pH 11.0, while flavanonols were indicated by orange-red. Chalcones and aurones were indicated by a red color at pH 3.0 and red-purple at pH 11.0, respectively.

Sterols and triterpens were investigated by dissolving 250 mg of each sample into 5 mL of chloroform. The samples were filtered and the insoluble material was used to identify saponins. Acetic anhydride (1 mL) and 60 *μ*L sulfuric acid (Liebermann-Burchard solution) were added to the soluble phase. A green color indicated free sterols, while a brown-red color indicated triterpens. To investigate saponins, 5 mL of distilled water was added to the precipitate and agitated for 3 min. Saponins were indicated by a persistent foam ring. 

Alkaloids were indicated as follows: ammonium hydroxide was added to 3 mL of latex fractions to pH 11.0 and ether-chloroform (3 + 1) was successively added (at 30, 20 and 10 mL) using a fractionator. The ether-chloroform phase was eliminated and the extracted alkaloids were filtered in 0.1 M HCl. The resulting material was divided in three parts and an equal volume of the alkaloid precipitation solutions (Hager, Mayer and Dragendorff) was added. Flocculated material indicated a positive reaction.

### 2.5. Infrared and ^1^H-NMR Spectroscopy

 Infrared analyses were performed using a Perkin Elmer spectrometer (model FT-IR Spectrum 1000). Potassium bromide (KBr) was used to prepare pastilles for the samples. Prior to ^1^H-NMR spectroscopy, the samples (25 mg/mL) were dissolved in deuterated chloroform (CDCl_3_) and methanol (CD30D). ^1^H-NMR spectra were recorded using a Bruker Spectrometer (model Advance DPX 500) at 500 MHz. Trimethyl silane (TMS) was added as an internal standard.

### 2.6. Chemical Detection of Cyclopeptides Using Tolidine and Ninhydrin

The fractions (10 mg/mL) were dissolved in methanol and applied to a thin layer chromatography (TLC) plate with silica gel 60 F254 (Merck). The peptide-containing fractions were detected with a Cl_2_/o-tolidine reagent, which indicates amide groups that are typically found in peptide bonds [13]. The ninhydrin reagent was used to indicate amino groups released by HCl hydrolysis in accordance with the methodology described by Tan and Zhou [14].

### 2.7. Biological Assays

#### 2.7.1. Animals

Adult male Wistar rats (180–200 g) were obtained from the Central Animal House of Universidade Federal do Ceará. The animals were maintained in a room with free access to water and commercial feed (Purina, Paulínia, SP, Brazil) at 25 (±3)°C and 70 (±5)% humidity until they were used for experiments. The animals were handled and experiments were performed in accordance with the standards described in the “Guide for the Care and Use of Laboratory Animals” of the National Research Council and submitted for approval by the local animal ethics committee (protocol number 24/09).

#### 2.7.2. MTT Assay

The latex fraction cytotoxicities were evaluated for four human tumor cell lines: HL-60 (human leukemia), Ovcar-8 (human ovarian adenocarcinoma), HCT-116 (human colon cancer), and SF-295 (human glioblastoma). The cultured cell viabilities were determined by reducing the yellow dye 3-(4,5-dimethyl-2-thiazolyl)-2,5-diphenyl-2H-tetrazolium bromide (MTT) to a blue formazan product, as previously described by Mosmann [15]. The cells were cultured in RPMI 1640 medium supplemented with 10% fetal bovine serum, 2 mM glutamine, 100 U/mL penicillin and 100 *μ*g/mL streptomycin and maintained at 37°C in 5% CO_2_. For the assay, cells were plated in 96-well plates (0.1 × 10^6^ cells/mL for adherent cells and 0.3 × 10^6^ cells/mL for suspended cells) and incubated to facilitate cell adhesion. Twenty-four hours later, fractions (100 *μ*L) were added to each well (0.39–25 *μ*g/mL). The mixtures were incubated for 72 hours, and thus the supernatant was replaced by fresh media with 10% MTT. The formazan product formed was dissolved in DMSO and the absorbance was measured at 595 nm (DTX-880, Beckman Coulter). Doxorubicin (0.009–5 *μ*g/mL) was used as a positive control.

#### 2.7.3. Brine Shrimp Lethality Assay

Latex fraction toxicity was further evaluated for an aquatic nontarget species (Artemia sp.) following the methodology described by Carvalho et al. [16]. Groups of ten Artemia sp. nauplii (24 hours old) received different concentrations (10–1,000 *μ*g·mL^−1^) of the extracts (100 *μ*L) in triplicate. After 24 hours, the number of living animals was recorded. A standard potassium dichromate solution (LD_50_ = 20 *μ*g·mL^−1^) was used as a positive control.

#### 2.7.4. Anti-Inflammatory Assay

The fractions were dissolved in 5% DMSO and the insoluble materials were removed through centrifugation (10 min; 10°C; and 10,000 g). Clean samples were intravenously injected into rats (*n* = 6, per group) at different doses (1.0; 5.0 or 10.0 mg/kg; and 0.2 mL) 30 minutes before the carrageenan stimulus (700 *μ*g/cavity and i.p.). The control animals received sterile saline. Four hours later, animals were sacrificed through Halothane inhalation, and the peritoneal cavities were washed with 10 mL of sterile saline with 5 UI/mL of heparin. The fluids were recovered for a total and differential cell count using light microscopy in accordance with Souza and Ferreira [17].

#### 2.7.5. Hemolytic Activity

Human erythrocytes (50 mL) were obtained from the Centre of Hematology and Hemotherapy of the State of Ceará (Ceará, Brazil). After three wash cycles in 150 mM NaCl, erythrocytes were suspended in a washing solution at 4%.

The latex fractions were dissolved in 5% DMSO at a final concentration of 4 mg/mL. A 100 *μ*L aliquot of each fraction was added to 100 *μ*L of PBS, and serial dilution was used to determine the final concentration range from 1 to 0.07 mg/mL. Finally, 100 *μ*L of erythrocytes (4%) were incubated in MicroAmp 96-well plates for 30 min at 37°C. The plate was then centrifuged (700 g for 10 min at 4°C) to remove the lysed cells. Hemoglobin content was determined using the supernatant through spectrophotometry at 540 nm and correlated with the erythrocyte lysate. The cells' maximum lysate values (100%) were determined by incubating an erythrocyte suspension with 0.1% (v/v) of Triton X-100. The experiments were performed in triplicate. The percentage of hemolysis (H) was calculated using the following equation: H = 100 × [(Op − Ob)/(Ot − Ob)], where Op is the density for a given fraction concentration, Ob is the PBS buffer density, and Ot is the triton X-100 positive control density. 

### 2.8. Statistical Analyses

All results are expressed as the mean ± S.E.M. The data were analyzed by one-way ANOVA for multiple comparisons, followed by Bonferroni's test in anti-inflammatory assays. *P* < 0.05 was considered statistically significant in all analyses. 

## 3. Results and Discussion

### 3.1. Phytochemical Profile

Latex from *C. procera* has been described as an important source of secondary metabolites [10]. In this study, the latex was fractionated and qualitatively evaluated. Phenols, tannins, saponins, and alkaloids were not detected through phytochemical screening. Nevertheless, compounds in these groups were reported for nonlatex organs and tissues in *C. procera* [1, 11, 18, 19]. However, compounds with such chemical characteristics were reported in latex for Euphorbiaceae, Convolvulaceae, Anacardiaceae, and Papaveraceae plants [20]. Therefore, *C. procera* (Apocynaceae) latex is chemically distinct. Steroids, flavonoids, and triterpens were detected in at least two distinct latex fractions (Table 1). Such a profile was previously reported for *C. procera* latex and other fractions [10, 21]. Triterpens (isoprene derivatives) are the most abundant and common molecules in natural rubbers [22].

**Figure 1:**
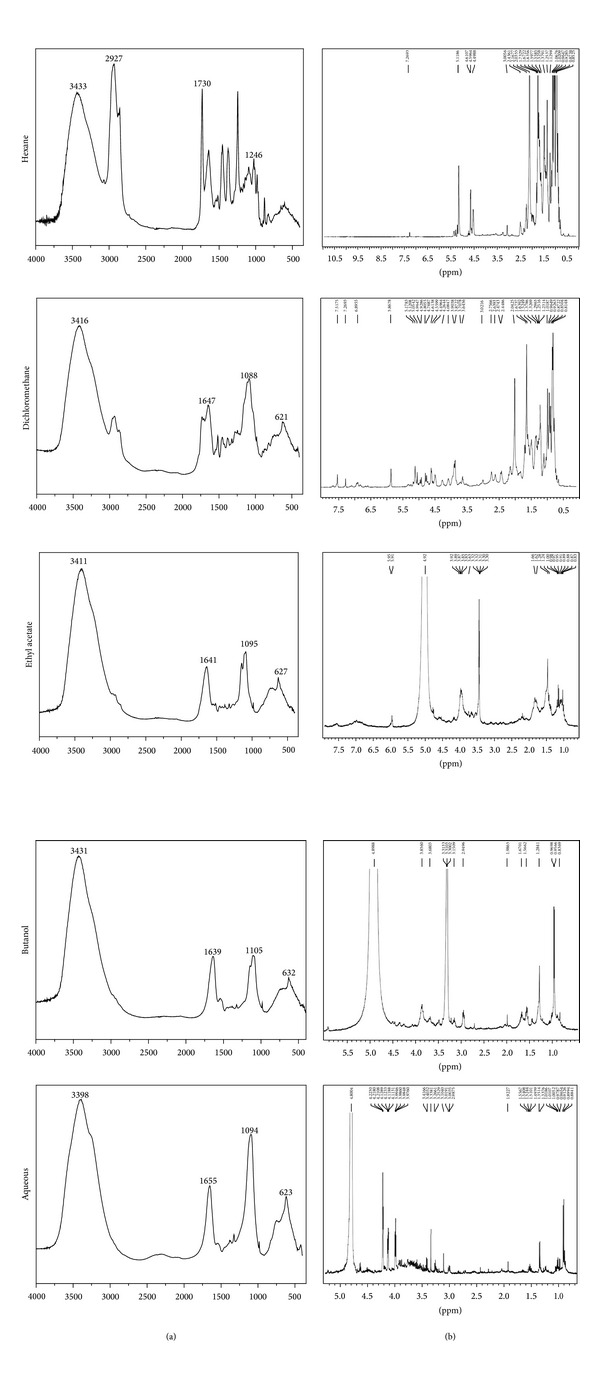
Fingerprinting analysis for *C. procera* latex fractions through infrared spectroscopy (left panel) and ^1^H-RMN (right panel).

Further analyses on the chemical diversity of latex fraction were performed using FT-IR and ^1^H-NMR spectroscopic techniques (Figure 1). The most hydrophobic fractions, hexane and dichloromethane, produced different FT-IR spectra which in turn were distinct from the ethyl acetate, butanol, and aqueous fractions, which produced almost identical spectra.

Hydroxyl groups (OH) with peaks at ~3400 cm^−1^ were in all of the fractions. The infrared spectra for hexane and dichloromethane presented peaks at 2961–2854 cm^−1^, which confirmed C–H stretching in methyl or methylene groups. The peaks at 1723 cm^−1^ showed C=O stretching or methyl group asymmetrical deformations/vibrations. Additional peaks at 1450 cm^−1^ (C–H deformation) and 1350 cm^−1^ (C–H deformation in CH_3_) were also observed. These prominent peak signals were decreased in the dichloromethane fraction.

Poly-isoprene, which is the precursor for rubber polymers, was detected in abundance for the hydrocarbon fractions as first described by Kalita and Saikia [22]. According to the hexane and dichloromethane IR spectra (Figure 1), *C. procera* (poly) isoprene is likely common to both hydrophobic fractions. This is consistent with the data in Table 1. 

The ethyl acetate, butanol, and aqueous infrared fraction peaks were detected at ~1645 cm^−1^. Thus, they were assigned to the double bond (C=C) and 1100–1080 cm^−1^ for C–O stretching. The infrared spectrum for the aqueous fraction produced signals at 1093 cm^−1^ (C–O stretching). This peak was stronger than in both the ethyl acetate and butanol fractions, which is expected given the higher polarity of the sample. These data are also supported by the data in Table 1, which is suggestive of steroids.

^1^H-NMR spectra in the high field region showed remarkably high proton levels (hexane and dichloromethane) assigned to aliphatic chain CH_3_ and CH_2_ groups. Residual protons that were similar were also observed in the ethyl acetate, butanol and aqueous spectra (Figure 1). ^1^H-NMR spectra for the dichloromethane fraction showed peaks at the middle-field region assigned to olefinic-like protons commonly observed in natural rubbers [22]. In the aqueous fraction, the peaks were primarily assigned to protons bound to oxygenated carbons. Peaks in the low-field region were observed both for hexane as well as dichloromethane and were likely aromatic molecules. The ^1^H-NMR results showed that dichloromethane was the most complex fraction with hydrogen chemical shifts in the low, middle and high fields. These findings are consistent with phytochemical screening (Table 1) showing triterpens, steroids, and flavonoids in the latex, which is also supported by the literature [10].

## 4. Toxicity

The cytotoxicity of *C. procera* latex fractions was investigated using the MTT assay in four tumor cell lines (HL-60, Ovcar-8, HCT-116 and SF-295). The more hydrophobic fractions (hexane, dichloromethane, and ethyl acetate) were cytotoxic to the tumor cells tested with LD_50_ values that ranged from 0.05 to 6.5 *μ*g/mL (Table 2). The hydrophilic fractions (butanol and aqueous) were not cytotoxic. Sawadogo et al. [23] reviewed West African plants with anticancer properties. Three anticancer cardenolides (2′′-Oxovoruscharin, uscharin and voruscarin) isolated from *C. procera* were reported as highly antiproliferative to different cancer cell lines (Hs683, U373, HCT-15, LoVo and A549); the latter two are in latex [10]. *C. procera* methanolic extract is cytotoxic to cancer cell lines and exhibits chemopreventive activity in vitro and in vivo in hepatocellular carcinoma [4]. More recently, studies by Juncker et al. [24] showed potential for UB450, which is a hemisynthetic cardenolide, to repress cancer cell proliferation and induce cell death.

Finally, Magalhães et al. [25] showed that ethyl acetate, acetone, and methanol stem extract from *C. procera* are promising due to in vitro antiproliferative activity on cancer cell lines. It should also be noted that selective cytotoxicity and in vivo anticancer properties have been reported in the latex protein fractions [26, 27]. The five fractions tested were not hemolytic for human erythrocytes at concentrations up to 1 mg/mL.

*C. procera* latex fraction toxicity was further examined using the brine shrimp lethality bioassay. As shown in Table 2, the LD_50_ values determined for dichloromethane and ethyl acetate suggested that these fractions were cytotoxic. These data are supported by in vivo studies that correlated bioactive plants constituents and toxicity [28]. Certain consistent reports in the literature also show that plant extracts with LD_50_ values lower than 250 *μ*g/mL are significantly toxic [29, 30]. The dichloromethane LD_50_ value was lower than cyclophosphamide (LD_50_ 16.3 *μ*g/mL), which was used as positive control in the study by Moshi et al. [31]. The LD_50_ values determined for the other fractions were higher and suggested no acute toxicity for such fractions.

## 5. Folk Use and Anti-Inflammation

Anti-inflammatory activity is among different uses for *C. procera *in folk medicine. In fact, numerous scientific reports have confirmed and extensively characterized its anti-inflammatory activity in different models [3, 32]. Dichloromethane, ethyl acetate, and aqueous fractions inhibited carrageenan-induced neutrophil migration in rats at the ratios 67%, 56%, and 72%, respectively (Table 3). 

Both the hexane and butanol fractions did not inhibit neutrophil migration. Kumar and colleagues have extensively demonstrated and characterized anti-inflammatory activity in aqueous and alcoholic *C. procera* latex extracts [33, 34]. Seddek et al. [35] studied water-soluble *C. procera* latex extract and demonstrated that it enhances iNOS gene expression as well as NO production in murine macrophages, which facilitate inflammatory and immune response effector cell activation. More recently, Tour and Talele [36] reported that both the chloroform and hydroalcoholic extracts from *C. procera* stem bark at 200 and 400 mg/kg exhibited anti-inflammatory activity, respectively. In addition, chloroform extract at 400 mg/kg exhibited a significant gastromucosal protective effect.

## 6. Cyclopeptides in Latex

**Figure 2:**
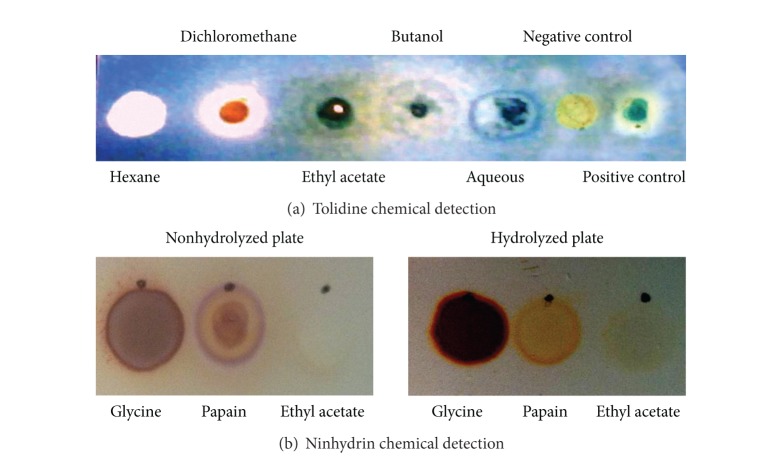
Cyclopeptides in ethyl acetate fraction from *C. procera *are confirmed by tolidine (a) and ninhydrin chemical detection (b).

Ethyl acetate, butanol, and aqueous fractions reacted positively with the Cl_2_/o-tolidine reagent (Figure 2), which indicates amide groups that are typically in peptide bonds according to Van Den Berg et al. [37]. 

Plants in different angiosperm families are notorious for accumulating peptides, primarily cyclic peptides (Annonaceae, Caryophyllaceae, Euphorbiaceae, Rubiaceae, and Violaceae) [38, 39]. Likewise, peptides/cyclopeptides have been described for laticiferous plants in the *Jatropha* genus, for which many isolated peptides were characterized (Integerrimides, curcacyclines, jatrophidin, pohlianin, podacycline, cyclogossines, chevalierins, and mahafacyclin) [40]. The cyclic peptide purified from the *Stephanotis floribunda* stem is the only report for such molecules in Asclepiadaceae members [41]. Thus, we examined the most promising ethyl acetate fraction for cyclopeptides. 

Many reports in the literature have shown that cyclopeptides from laticiferous plants have been isolated from the ethyl acetate fraction [40, 42, 43]. Thus, considering that this *C. procera* fraction was cytotoxic and anti-inflammatory and reacted positively for amide groups, it was reexamined using ninhydrin. Ninhydrin reacts with free amino groups (typically in proteins). As shown in Figure 2, ninhydrin reacted positively with papain (protein) and glycine (free amino acid), which were the positive controls, and did not react with the ethyl acetate fraction, which suggests no free/accessible amino groups in the latter sample. However, after chemical hydrolysis by 6 M HCl, the ethyl acetate fraction reacted positively with ninhydrin, which suggests free amino groups. Therefore, it was concluded that cyclopeptides are in this latex fraction. 

Cyclopeptides with cytotoxicity and anticancer properties were reported in the roots and other tissues for different plants [44–47]. The study by Mongkolvisut et al. [48] is the only manuscript that has reported latex cyclopeptides (Integerrimides A and B) with a proliferative effect on human IPC-298 melanoma cells and a migration effect on human Capan II pancreatic carcinoma cells. More recently, studies have reported three new cyclopeptides that exhibited anti-inflammatory activity in vitro using the J774.1 macrophage model [49]. These results imply that cyclopeptides from *C. procera* latex are involved in the ethyl acetate fraction pharmacological properties. Cyclopeptide detection is especially relevant because, until now, attempts to investigate these low molecular cyclic amino acid sequences in *C. procera* have failed. Purification and structural analyses for such molecules are currently the greatest challenge in the field.

## 7. Conclusions

The work herein shows that the *C. procera* latex dichloromethane and ethyl acetate fractions exhibit potential toxicity both in vitro and in vivo as well as anti-inflammatory properties. *C. procera* latex cyclopeptides will be the next materials used in the investigations for compounds involved in the latex pharmacological properties.

## Figures and Tables

**Table 1:** Preliminary phytochemical screening of fractions of *C. procera* latex.

Fractions	Yield (%)	Phytochemicals						
		Phenols	Tannins	Flavonoids	Steroids	Triterpens	Saponins	Alkaloids
Hexane	49.4	−	−	−	+	+	−	−
Dichloromethane	5.2	−	−	+	+	+	−	−
Ethyl acetate	2.0	−	−	+	+	−	−	−
Butanol	2.1	−	−	−	+	−	−	−
Aqueous	41.1	−	−	−	+	−	−	−

Positive (+) sign indicates positive reaction of the compound tested while negative sign (−) indicates the absence.

**Table 2:** Evaluation of toxicity potential of fractions of *C. procera* latex against cell lines and Brine shrimp nauplii.

Fractions	Sample (LC_50_ *µ*g·mL^−1^)				
	MTT assay				Brine shrimp assay
	HL-60	Ovcar-8	HCT-116	SF-295	
Hexane	2.9	6.5	3.8	3.2	781.5 ± 31.2
	(2.4–3.4)	(5.6–7.6)	(3.5–4.1)	(2.7–3.8)	(721.2–843.3)
Dichloromethane	0.05	0.17	0.11	0.12	10.9 ± 0.9
	(0.04–0.06)	(0.14–0.2)	(0.1–0.13)	(0.1–0.14)	(9.3–12.7)
Ethyl acetate	1.8	3.9	1.7	1.8	65.7 ± 4.6
	(1.5–2.1)	(3.4–4.4)	(1.6–1.8)	(1.6–1.9)	(57.3–75.3)
Butanol	>100	>100	>100	>100	237.3 ± 22.3
					(195.2 – 284.7)
Aqueous	>100	>100	>100	>100	712.5 ± 39.9
					(636.2–792.6)
Doxorubicin	0.02	1.36	0.01	0.24	—
	(0.01–0.02)	(0.98–1.89)	(0.01–0.02)	(0.17–0.36)	
Potassium dichromate	—	—	—	—	20

*Values are expressed as mean ± S.E.M. Values in parenthesis represent *P* < 0.05.

**Table 3:** Inhibitory effect of fractions of *C. procera* latex on carrageenan-induced peritonitis model.

Fractions	Neutrophils × 10^3^/mL (control group)	Neutrophils × 10^3^/mL (10 mg/kg)	Inhibition (%)
Hexane	2191 ± 332.0	1422 ± 265.9	—
Dichloromethane	2191 ± 332.0	720.4 ± 150.3	67
Ethyl acetate	7599 ± 383.5	3369 ± 585.6	56
Butanol	2869.25 ± 239.5	1409 ± 557.7	—
Aqueous	7515.8 ± 423.3	2118 ± 151.2	72

*Values are expressed as mean ± S.E.M (*n* = 5; *P* < 0.05; ANOVA followed by Bonferroni's test).

## Abbreviations

FT-IR: fourier transform infrared spectroscopy
NO: nitric oxide.

## References

[1] Chundattu SJ, Agrawal VK, Ganesh N (2011). Phytochemical investigation of *Calotropis procera*. *Arabian Journal of Chemistry*.

[2] Alencar NMN, Oliveira JS, Mesquita RO (2006). Pro- and anti-inflammatory activities of the latex from *Calotropis procera* (Ait.) R.Br. are triggered by compounds fractionated by dialysis. *Inflammation Research*.

[3] Kumar VL, Arya S, Govil JN (2006). Medicinal uses and pharmacological properties of *Calotropis procera*. *Recent Progress in Medicinal Planta*.

[4] Choedon T, Mathan G, Arya S, Kumar VL, Kumar V (2006). Anticancer and cytotoxic properties of the latex of *Calotropis procera* in a transgenic mouse model of hepatocellular carcinoma. *World Journal of Gastroenterology*.

[5] Oh SK, Kang H, Shin DH, Yang J, Han KH (2000). Molecular cloning and characterization of a functional cDNA clone encoding isopentenyl diphosphate isomerase from Hevea brasiliensis. *Journal of Plant Physiology*.

[6] Parijs JV, Broekaert WF, Goldstein IJ, Peumans WJ (1991). Hevein: an antifungal protein from rubber-tree (*Hevea brasiliensis*) latex. *Planta*.

[7] Elgamal MHA, Hanna AG, Morsy NAM (1999). Complete ^1^H and ^13^C signal assignments of 5*α*-cardenolides isolated from *Calotropis procera* R. BR. *Journal of Molecular Structure*.

[8] Freitas DTC, Nogueira FCS, Vasconcelos IM, Oliveira JTA, Domont GB, Ramos MV (2011). Osmotin purified from the latex of *Calotropis procera*: biochemical characterization, biological activity and role in plant defense. *Plant Physiology and Biochemistry*.

[9] Ramos MV, Grangeiro TB, Freire EA (2010). The defensive role of latex in plants: detrimental effects on insects. *Arthropod-Plant Interactions*.

[10] Meena AK, Yadav AK, Niranjan US (2010). A review on *Calotropis procera* Linn and its ethnobotany, phytochemical, pharmacological profile. *Drug Invention Today*.

[11] Murti Y, Yogi D B (2010). Pathak Pharmagnostic standartization of leaves of *Calotropis procera* (Ait.) R. Br. (Asclepiadaceae). *International Journal of Ayurveda Research*.

[12] Matos FJA (1997). *Introdução A Fitoquímica Experimental*.

[13] Horsten SFAJ (1995). *Cyclic Peptides in the Genus Jatropha (Euphorbiaceae)*.

[14] Tan NH, Zhou J (2000). Application of a new TLC chemical method for detection of cyclopeptides in plants. *Chinese Science Bulletin*.

[15] Mosmann T (1983). Rapid colorimetric assay for cellular growth and survival: application to proliferation and cytotoxicity assays. *Journal of Immunological Methods*.

[16] Carvalho AFU, Melo VM, Aguiar LMBA, Matos FJA (1998). Avaliação da toxicidade de extratos de plantas medicinais através de bioensaio com Artemia salina. *Leach Ciência E Cultura*.

[17] Souza GEP, Ferreira SH (1985). Blockade by antimacrophage serum of the migration of PMN neutrophils into the inflammed peritoneal cavity. *Agents Actions*.

[18] Mainasara MM, Aliero BL, Aliero AA, Dahiru SS (2011). Phytochemical and antibacterial properties of *Calotropis Procera* (Ait) R. Br. (Sodom Apple) fruit and bark extracts. *International Journal of Modern Botany*.

[19] Doshi H, Satodiya H, Thakur MC, Parabia F, Khan A (2011). Phytochemical screening and biological activity of *Calotropis Procera* (Ait). R.Br. (Asclepiadaceae) against selected bacteria and *Anopheles stephansi* Larvae. *International Journal of Plant Research*.

[20] Konno K (2011). Plant latex and other exudates as plant defense systems: roles of various defense chemicals and proteins contained therein. *Phytochemistry*.

[21] Shaker KH, Morsy N, Zinecker H, Imhoff JF, Schneider B (2010). Secondary metabolites from *Calotropis procera* (Aiton). *Phytochemistry Letters*.

[22] Kalita D, Saikia CN (2004). Chemical constituents and energy content of some latex bearing plants. *Bioresource Technology*.

[23] Sawadogo WR, Schumacher M, Teiten MH, Dicato M, Diederich M (2013). Traditional West African pharmacopeia, plants and derived compounds for cancer therapy. *Biochemical Pharmacology*.

[24] Juncker T, Schumacher M, Dicato M, Diederich M (2009). UNBS1450 from *Calotropis procera* as a regulator of signaling pathways involved in proliferation and cell death. *Biochemical Pharmacology*.

[25] Magalhães HIF, Ferreira PMP, Moura ES (2010). In vitro and in vivo antiproliferative activity of calotropis procera stem extracts. *Anais da Academia Brasileira de Ciencias*.

[26] Oliveira JS, Costa-Lotufo LV, Bezerra DP (2010). In vivo growth inhibition of sarcoma 180 by latex proteins from *Calotropis procera*. *Naunyn-Schmiedeberg’s Archives of Pharmacology*.

[27] Soares de Oliveira J, Pereira Bezerra D, Teixeira de Freitas CD (2007). In vitro cytotoxicity against different human cancer cell lines of laticifer proteins of *Calotropis procera* (Ait.) R. Br. *Toxicology in Vitro*.

[28] Morshed MA, Uddin A, Rahman A (2011). In vitro antimicrobial and cytotoxicity screening of *Terminalia arjuna* ethanol extract. *International Journal of Biosciences*.

[29] Rieser MJ, Gu Z-M, Fang X-P, Zeng L, Wood KV, McLaughlin JL (1996). Five novel mono-tetrahydrofuran ring acetogenins from the seeds of *Annona muricata*. *Journal of Natural Products*.

[30] Meyer BN, Ferrigni NR, Putnam JE (1982). Brine shrimp: a convenient general bioassay for active plant constituents. *Planta Medica*.

[31] Moshi MJ, Cosam JC, Mbwambo ZH, Kapingu M, Nkunya MHH (2004). Testing beyond ethnomedical claims: brine shrimp lethality of some Tanzanian plants. *Pharmaceutical Biology*.

[32] Kumar VL, Roy S (2007). Calotropis procera latex extract affords protection against inflammation and oxidative stress in Freund’s complete adjuvant-induced monoarthritis in rats. *Mediators of Inflammation*.

[33] Kumar VL, Roy S (2009). Protective effect of latex of *Calotropis procera* in Freund’s Complete Adjuvant induced monoarthritis. *Phytotherapy Research*.

[34] Kumar VL, Chaudhary P, Ramos MV, Mohan M, Matos MPV (2011). Protective effect of proteins derived from the Latex of *Calotropis procera* against inflammatory hyperalgesia in monoarthritic rats. *Phytotherapy Research*.

[35] Seddek ALS, Mahmoud ME, Shiina T (2009). Extract from *Calotropis procera* latex activates murine macrophages. *Journal of Natural Medicines*.

[36] Tour N, Talele G (2011). Anti-inflammatory and gastromucosal protective effects of *Calotropis procera* (Asclepiadaceae) stem bark. *Journal of Natural Medicines*.

[37] Van Den Berg AJJ, Horsten SFAJ, Kettenes-Van Den Bosch JJ (1995). Curcacycline A—a novel cyclic octapeptide isolated from the latex of *Jatropha curcas* L. *FEBS Letters*.

[38] Tan N-H, Zhou J (2006). Plant cyclopeptides. *Chemical Reviews*.

[39] Picchi DG, Altei WF, Saito MS, Bolzani VS, Cilli EM (2009). Peptídeos cíclicos de biomassa vegetal: características, diversidade, biossíntese, e atividades biológicas. *Quimica Nova*.

[40] Zhang X-P, Zhang M-L, Su X-H, Huo C-H, Gu Y-C, Shi Q-W (2009). Chemical constituents of the plants from genus Jatropha. *Chemistry and Biodiversity*.

[41] Yoshikawa K, Tao S, Arihara S (2000). Stephanotic acid, a novel cyclic pentapeptide from the stern of *Stephanotis floribunda*. *Journal of Natural Products*.

[42] Baraguey C, Auvin-Guette C, Blond A (1998). Isolation, structure and synthesis of chevalierins A, B and C, cyclic peptides from the latex of Jatropha chevalieri. *Journal of Chemical Society Perkins*.

[43] Van Den Berg AJJ, Horsten SFAJ, Kettenes-Van Den Bosch JJ (1996). Podacycline A and B, two cyclic peptides in the latex of Jatropha podagrica. *Phytochemistry*.

[44] Cozzolino R, Palladino P, Rossi F, Calì G, Benedetti E, Laccetti P (2005). Antineoplastic cyclic astin analogues kill tumour cells via caspase-mediated induction of apoptosis. *Carcinogenesis*.

[45] Wélé A, Zhang Y, Ndoye I, Brouard J-P, Pousset J-L, Bodo B (2004). A cytotoxic cyclic heptapeptide from the seeds of *Annona cherimola*. *Journal of Natural Products*.

[46] Hsieh P-W, Chang F-R, Wu C-C (2004). New cytotoxic cyclic peptides and dianthramide from *Dianthus superbus*. *Journal of Natural Products*.

[47] Napolitano A, Rodriquez M, Bruno I (2003). Synthesis, structural aspects and cytotoxicity of the natural cyclopeptides yunnanins A, C and phakellistatins 1, 10. *Tetrahedron*.

[48] Mongkolvisut W, Sutthivaiyakit S, Leutbecher H (2006). Integerrimides A and B, cyclic heptapeptides from the latex of Jatropha integerrima. *Journal of Natural Products*.

[49] Chuang P-H, Hsieh P-W, Yang Y-L (2008). Cyclopeptides with anti-inflammatory activity from seeds of *Annona montana*. *Journal of Natural Products*.

